# Chronic neural activity recorded within breast tumors

**DOI:** 10.1038/s41598-020-71670-y

**Published:** 2020-09-09

**Authors:** Grant A. McCallum, Jay Shiralkar, Diana Suciu, Gil Covarrubias, Jennifer S. Yu, Efstathios Karathanasis, Dominique M. Durand

**Affiliations:** 1 Department of Biomedical Engineering, Case Western Reserve University, Cleveland, OH USA; 2 Case Comprehensive Cancer Center, Case Western Reserve University, Cleveland, OH USA; 3 Department of Radiation Oncology, Taussig Cancer Institute, Cleveland Clinic, Cleveland, OH USA; 4 Department of Cancer Biology, Lerner Research Institute, Cleveland Clinic, Cleveland, OH USA

**Keywords:** Breast cancer, Autonomic nervous system, Extracellular recording

## Abstract

Nerve fibers are known to reside within malignant tumors and the greater the neuronal density the worse prognosis for the patient. Recent discoveries using tumor bearing animal models have eluded to the autonomic nervous system having a direct effect on tumor growth and metastasis. We report the first direct and chronic in vivo measurements of neural activity within tumors. Using a triple-negative mammary cancer mouse model and chronic neural interface techniques, we have recorded neural activity directly within the tumor mass while the tumor grows and metastasizes. The results indicate that there is a strong connection between the autonomic nervous system and the tumor and could help uncover the mechanisms of tumor growth and metastasis.

## Introduction

An increasing amount of evidence supports the autonomic nervous system’s role in the etiology and evolution of solid tumors, including prostate, pancreas and breast. Solid tumors were once believed to lack innervation. Recent scientific articles indicate that nerve fibers infiltrate primary tumors through neurogenesis^1–3^. In breast and prostate cancers, increasing nerve densities are associated with more aggressive tumor grades and poor patient survival^4,5^. Signaling molecules such as neurotrophins, neuropeptides, axon guidance molecules and neurotransmitters are traditionally associated with nervous system function have also been implicated in cancer and may play an important role in promoting cancer outgrowth and expansion by mediating the reciprocal cross-talk between nerves and cancer cells, particularly in the context of the tumor microenvironment^6–8^.

Neurotransmitters were first shown to promote the migration of cancer cells in culture^9,10^ and a landmark study using genetically engineered mice revealed that sympathetic nervous system signaling through the neurotransmitter norepinephrine, can promote early prostate cancer development, while the parasympathetic branch of the nervous system, signaling through the acetylcholine neurotransmitter promotes a metastatic phenotype. Importantly, blocking neurotransmitter activity in this study inhibited prostate cancer development and progression^5^. However, the two branches of the autonomic nervous system (parasympathetic and sympathetic) appear to have different effects on tumor progression based on the cancer type. Increased sympathetic branch activity was found responsible for early prostate tumor growth, however, it is found to promote metastasis in breast cancer^11^. Elimination of sympathetic branch activity by chemical sympathectomy in rats with fibrosarcoma showed decreased tumor incidence and prolonged survival. In mice with melanoma, sympathectomy delayed development of tumors and prolonged survival. In the same study, no effect was seen in animals with parasympathetic activity eliminated via subdiaphragmatic vagotomies compared to their control groups^12^. However, in gastric tumors, reduction of parasympathetic branch activity via anterior or posterior subdiaphragmatic vagotomy actually suppressed tumorigenesis^13^.

Similar studies with breast cancer models reveal a different conclusion. Denervation of afferent vagus nerve sensory fibers through chemical means or through unilateral cervical vagotomies result in enhanced metastasis^14,15^. Chemical activation of the parasympathetic branch decreased breast cancer metastasis^16^. As mentioned above, increased sympathetic branch activity enhanced metastasis of primary breast cancer. Based on these breast cancer studies, increased vagus nerve activity to the brain can transmit information about precancerous cells via tumor-associated proinflammatory cytokines. Likewise, descending cholinergic-macrophage, vagal-dependent, anti-inflammatory routes slow or even prevent tumorigenesis by inhibition of tumor-associated proinflammatory cytokines^17^. It is generally known that the sympathetic branch has higher levels of activity under stressful conditions commonly known as the fight-or-flight response. Population studies have shown the protective role of beta-blockers prescribed during breast cancer treatment, against cancer progression^18,19^ and increased sympathetic activity in animal studies showed increased, stress-induced tumor progression^20^. These studies provide a starting point to understand the autonomic nervous system’s influence on breast cancer progression. However, none of these studies have reported direct recordings and/or quantified the neural activity within the solid tumors to ascertain whether these neural fibers are active and, if so, what neural signaling patterns are occurring within the solid tumor during its development through metastasis.

Here, we report the recorded chronic, in situ, electrical activity within a solid peripheral mammary tumor. Furthermore, using chronic neural interface techniques, ultra-low noise recording electronics and bioluminescence imaging (BLI) enabled us to record the daily electrical activity and detect when metastasis occurred. We have discovered a consistent and unique electrical activity pattern in a triple-negative, mammary cancer mouse model.

## Results

### Electrogram recordings within mammary tumors: experimental setup

**Figure 1:**
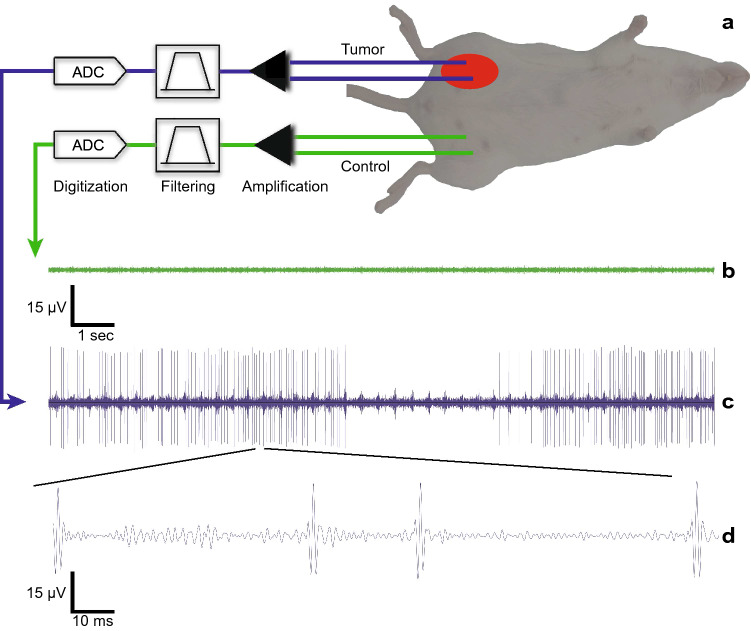
Experimental setup and resulting electrograms of chronic recordings from tumor bearing mice. (**a**) Two microwire electrodes (each 125 µm diameter) are implanted in the mammary tumor mass and two additional microwires are implanted in the contralateral side to act as a control during the electrical recording sessions. The electrodes are connected to a custom differential recording system that amplifies, filters and digitizes the electrical recording data that is saved to a laptop computer. (**b**) The resulting electrogram recordings from the control electrodes showing no activity. (**c**) Simultaneous electrogram recordings from within the tumor mass showing a high-level of electrical activity. (**d**) Magnified section of the tumor activity showing the classic spike waveform. These spike waveforms are individually counted for each 30-min recording session performed daily throughout this study. Activity is segmented into 10 min sections for analysis.

The murine 4T1 model represents a model of triple-negative mammary cancer, which develops metastasis in immunocompetent mice at organs reminiscent of those observed in human patients. Female BALB/cJ mice were inoculated with 0.5 × 10^6^ 4T1-luc-GFP cells orthotopically in the no. 9 mammary fat pad. This cell line stably expresses firefly luciferase (luc) and green fluorescent protein (GFP) to allow noninvasive tracking and quantification of the cells in vivo and in histology^21–23^. The tumor masses grew until they reached 4 mm in diameter which was roughly 10 days, post-inoculation. As shown in Fig. 1a and Supplementary Fig. 1, the distal ends of two microwire electrodes were chronically implanted in the tumor mass and two microwire electrodes were implanted on the contralateral side and acted as a control signal when recording the neural activity. All four proximal microwire ends were subcutaneously tunneled and soldered to a fixed, percutaneous electrical connector port on the dorsal side of the animal (Supplementary Fig. 2).

**Figure 2:**
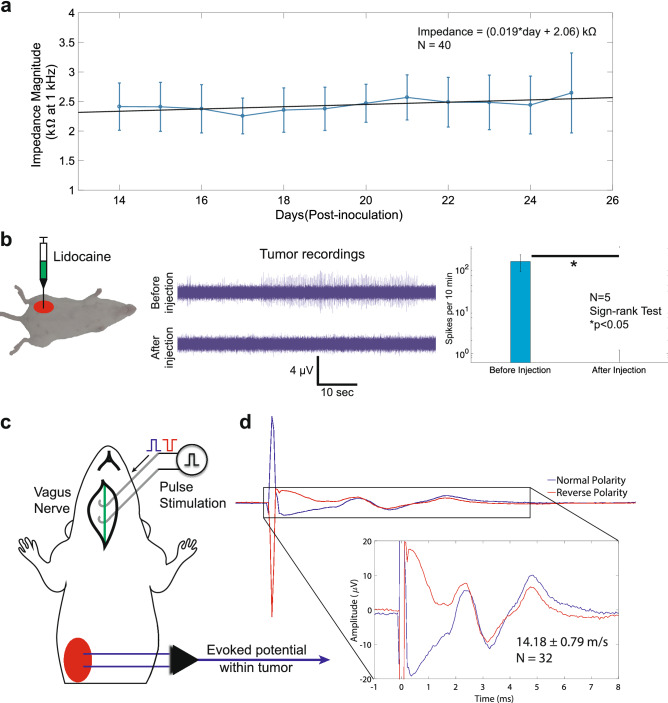
Stability of chronic interface electrode impedance and acute experiments to block and evoke electrical activity with the tumor mass. (**a**) Microwire electrode impedance magnitude at 1 kHz for 40 total implanted electrodes (10 mice with 4 electrodes each) measured daily over a 12-day period. Linear regression was performed on the data and a t-test on the slope of the resulting regression line revealed the slope was not significantly different from zero (*p* = 0.973) indicating a stable recording interface during the implant period. (**b**) Left: While under anesthesia, 0.5 mL of 2% Lidocaine injected directly into the tumor mass. Middle: Electrogram recording waveforms showing the electrical spike activity before injection and the elimination of the activity after injection. Right: Experiment was performed on five mice (n = 5) which showed a significant decrease in recorded electrical activity after the injection was administered (*p* < 0.05, sign-rank test). (**c**) A section of the right cervical vagus nerve in two mice were surgically exposed and isolated from surrounding tissue and placed on two hook electrodes. Repeated current simulation pulses (n = 8 normal polarity; n = 8 reverse polarity for each mouse, 0.25 Hz, 100 µs pulse width, 6 mA amplitude) were delivered to the nerve while simultaneously recording from within the tumor mass. (**d**) The evoked potentials were captured from within the tumor confirming that a neural connection exists between the brain and the tumor via the vagus nerve. In addition, the average neural conduction velocity (n = 32) was determined to be 14.18 ± 0.79 m/s which matches previously determined Aδ mouse afferent fiber types associated with transmitting pain information from the periphery to the brain.

Following a two-day rest period from electrode implantation, a custom, ultra-low noise neural recording amplifier board was connected directly to the percutaneous port to perform daily (Supplementary Fig. 3), simultaneous recordings from within both the tumor mass and the control channel for 30 consecutive minutes while the animal was anesthetized. The differential neural recording system performed the signal amplification, filtering for the desired neural bandwidth, and signal digitization (Supplementary Fig. 4) to be saved on a laptop computer^24^. Figure 1b,c and Supplementary Movie 1 show an example electrogram waveform recording for the control channel, tumor channel and the tumor channel with audio, respectively. The lack of activity recorded in the control channel eliminates the possibility of the tumor channel activity being motion or environmental noise artifacts since they would be present in both channels. To our knowledge, this is the first direct recording of direct electrical activity in peripheral tumors and confirms that certain tumor types are indeed electrically active and functional. Figure 1d is a magnified view of the tumor channel recording clearly identifying the classic spike waveforms associated with bioelectric activity. Using standard neural spike sorting software, these spike events were counted during each recording session throughout the course of the study. If electrical activity was detected simultaneously in both channels, the tumor channel activity was not counted during this time period. This was done to eliminate any false-positive events counted as tumor activity.

**Figure 3:**
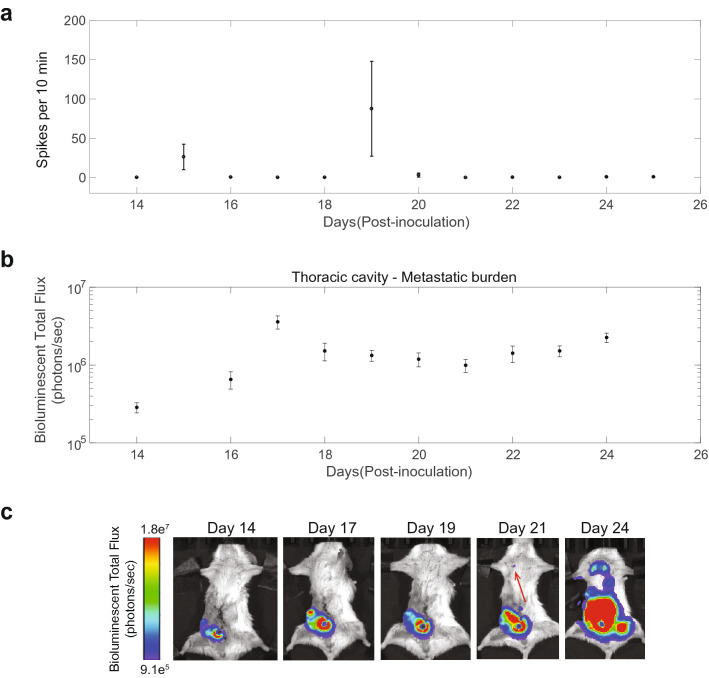
Single-mouse, chronic electrical recording results from within the breast tumor mass with the detection of metastasis using bioluminescence imaging. (**a**) Representative chronic electrical activity from a single mouse over a 12-day period that shows the two unique and consistent peak activity days found among all subjects in the experimental group (n = 10). The “First Peak” and “Second Peak” occurring at days 15 and 19 post-inoculation, respectively. (**b**) The bioluminescent signal of the thoracic cavity is recorded and displayed as a progression over time to monitor 4T1 cell expansion. Between day 14 and 17, a tenfold increase in bioluminescent total flux [photons/sec] indicating the onset of metastatic outgrowth. Data is represented as mean ± SEM (n = 5). (**c**) Representative images from a single mouse showing the daily bioluminescence imaging to monitor primary tumor growth and determine when metastasis to the lungs is detected as shown by the red arrow on day 21.

**Figure 4:**
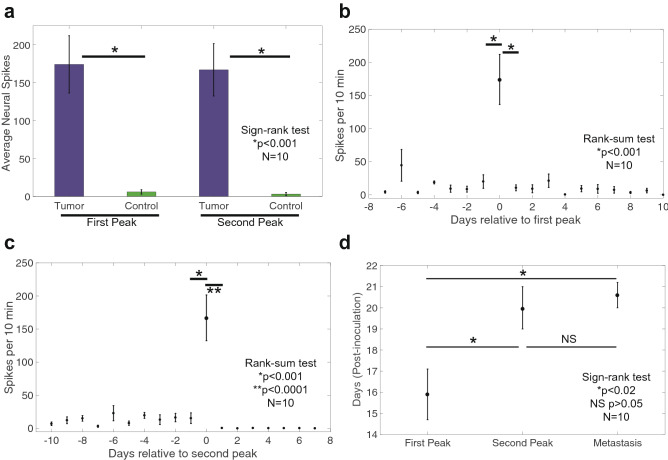
Chronic electrical recording results from within the breast tumor mass. (**a**) Comparing the average electrical spike counts on both the first and second peak activity days compared to their respective control channel activity shows a significant increase in activity within the tumor mass on both days (*p* < 0.001, sign-rank test, n = 10). (**b**) Taking all the subject’s chronic electrical spike counts and lining them up on the first peak day (day = 0), there is a significant reduction in the amount of activity in the day preceding (day = -1) and following (day =  + 1) the first peak day (*p* < 0.001, rank-sum test, n = 10). (**c**) Similarly, lining up all the chronic electrical spike counts for the second peak day shows a significant reduction in activity in the previous day (*p* < 0.001, rank-sum test, n = 10) and an even more significant reduction in activity on the following day of the second peak (*p* < 0.0001, rank-sum test, n = 10) in which the activity within the tumor mass resembles that of the control channel starting from day + 1. (**d**) Summary of the occurrences of the average first peak, second peak and metastasis detection (n = 10) are on days 16, 20 and 20.5, respectively. A significant difference between the days of the first peak and second peak and also between the first peak and the detection of metastasis (*p* < 0.02, sign-rank test, n = 10). However, there was no significant difference in the occurrence of the second peak activity day and metastasis detection (*p* > 0.05, sign-rank test, n = 10).

### Validating neural activity using acute chemical and electrical means

To further investigate the validity of the results and that the recorded signals within the tumor are truly neural in origin, we carried out the following experiments depicted in Fig. 2: tested the stability of the neural interface electrodes by measuring the impedance magnitude over time; administered lidocaine, a well-known chemical agent to block neural activity, and elicited a neural response within the tumor mass using electrical stimulation. Figure 2a shows the microwire electrode impedance magnitude measurements at 1 kHz over a 12 day, post-implant period. Data was collected from 10 different subjects each with four implanted microwire electrodes (n = 40). The average impedance value over the 12 days was 2.50 ± 0.21 kΩ (mean ± SD). Linear regression was performed on the data and a t-test was run on the regression line slope. The results indicate that the slope is not significantly different from zero (*p* = 0.973) indicating a stable electrode impedance magnitude over the 12-day recording period. High electrode impedance generates increased thermal noise leading to high amplitude baseline recording levels which can mask neural spikes from being detected. Here we show the electrodes have a low and stable impedance values in the frequency range of interest during the entire implant duration. At each subject’s terminal endpoint, it was visually confirmed that the implanted tumor electrodes were still within the tumor mass.

Lidocaine is an amide local anesthetic and is widely known to produce a reversible nerve block by altering the signal conduction in neurons by prolonging the inactivation of the fast voltage-gated Na^+^ channels in the neuronal cell membrane responsible for action potential propagation^25^. Neural recordings were started and activity was recorded for 10 consecutive minutes before a dose of lidocaine was injected directly into the tumor mass as shown in Fig. 2b. The neural recording continued for an additional 10 min and the electrogram recordings obtained before and after injection clearly show the elimination of the recorded activity. Analysis on the results obtained from five subjects indicate a statistically significant (*p* < 0.05) reduction in both the spike count activity and overall recorded root mean square (RMS) values within the tumor when comparing the activity before and after the lidocaine injection. No significant changes in activity in the control channel were observed.

To determine if the recorded activity within the tumor could be generated by the parasympathetic fibers innervating the tumor, acute evoked potential experiments on two mice were performed. As shown in Fig. 2c, while under anesthesia, a portion of the cervical vagus nerve was surgically exposed and isolated from the carotid artery. The exposed vagus nerve was placed on two hook electrodes and connected to a pulse stimulation unit. Monophasic current pulses of both polarities were delivered to the nerve and, simultaneously, tumor recordings were performed to capture the evoked response resulting from the current stimulation pulses. Figure 2d shows the resulting averaged electrogram responses from repeated trials with the large amplitude stimulation artifacts followed by the evoked potential roughly 3.3 ms after the stimulus was applied. The separation distance between the stimulation electrode and the recording electrode was approximately 5 cm resulting in a measured neural conduction velocity of 14.18 ± 0.79 m/s (mean ± SD). No evoked potential responses were captured in the control channel recordings.

The results from these acute experiments indicate that the electrical activity is neural in nature and show that vagal fibers innervate the tumor. They also suggest that the firing pattern within the tumor mass could be related to the tumor growth and metastasis.

### Chronic recordings of nerve activity within mammary tumors

Therefore, we tested the relationship between neural activity in the tumor and metastasis detection by performing chronic recording and bioluminescence imaging to quantify the neural activity within the tumor as it grew and metastasis was detected using BLI. Figure 3a is a representative plot of the daily neural spike activity from one tumor bearing mouse over a 12-day period. Consistent in all 10 mice recorded from, two peaks of high-level electrical activity were observed and are referred to as “First Peak” and “Second Peak”. Figure 3b shows the longitudinal BLI signal in the thoracic region and quantification of 4T1 cancer cells in the lungs as measured by flow cytometry (Supplementary Fig. 5). BLI was also performed to track the primary tumor progression and identify when detectable metastasis to the lungs occurred by BLI (red arrow in Fig. 3c). It should be noted that the disease has already spread to the liver and lungs on day 10 and formed early micrometastasis, it is still undetectable at that time using BLI. However, at the later time point, those early colonies have now become tumor lesions that can be comfortably detected by BLI.

**Figure 5:**
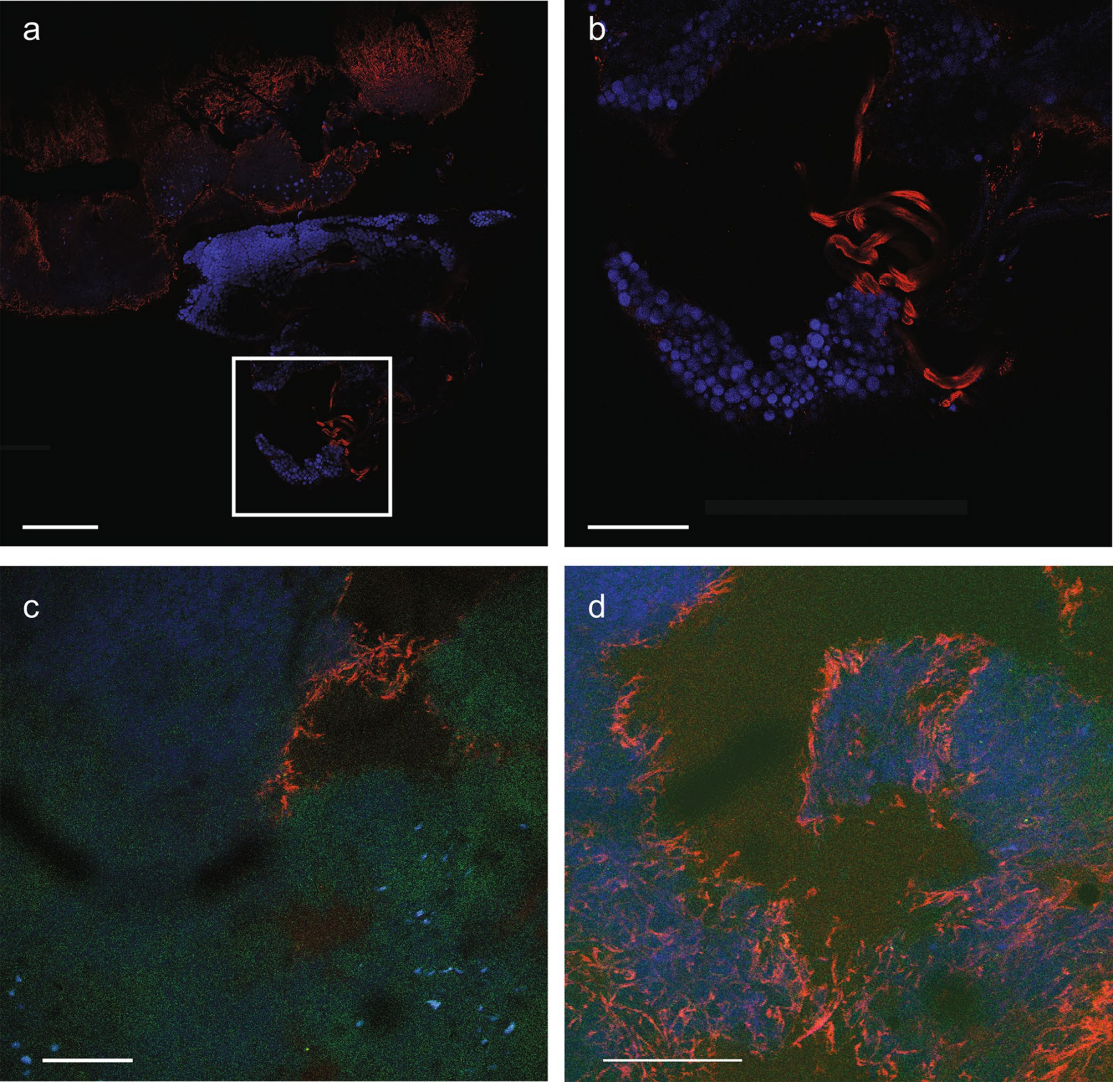
Histological analysis of 6-day, post-inoculation breast tumor mass. In a 1 mm thick tumor slice**,** nuclei were visualized by 4′,6-diamidino-2-phenylindole (DAPI, blue), neural fibers were visualized by neurofilament (NF, red) and cancer cells with Green Fluorescent Protein (GFP, green). Confocal microscopy was used to capture the images at different levels of the tissue. (**a**) Large scale composite image (DAPI + NF) of the tumor mass [scale bar = 1 mm] with most of the neural fibers being present near the periphery of the tumor. (**b**) Magnified composite image (DAPI + NF) of box in (A) which shows the neural fibers coursing through the tumor tissue [scale bar = 400 µm]. (**c**) Composite image (DAPI + NF + GFP) showing the prevalence of cancer cells and the nerve fibers embedded within [scale bar = 250 µm, 10x]. (**d**) Composite image (DAPI + NF + GFP) at a different level of the tumor tissue showing a higher concentration of nerve fibers among the cancer cells [scale bar = 250 µm, 20x].

Comparison of the recorded tumor activity to the control side activity during both peak days is shown in Fig. 4a. For both peak days, there is a significant difference (*p* < 0.001) in the number of spikes recorded indicating the tumor microenvironment is more highly active compared to the normal neural activity on the control side. In order to determine how fast the activity was changing during first and second peak, the data were normalized to the day of occurrence of the first peak (Fig. 4b) and second peak (Fig. 4c). We then compared the peak activity to the day preceding it (Day -1) and the day following it (Day + 1). The results show that the recorded neural activity during the day preceding and following each peak day was highly significant (*p* < 0.001 and *p* < 0.0001, respectively) indicating a rapid switch in activity within the tumor on the peak days.

We measured the day of the first detection of metastasis by BLI and plotted it on the same graph with the first and second peak (Fig. 4d). For all 10 mice, the first peak and second peak occurred at day 15.9 ± 3.7, 20.0 ± 3.3, respectively, and metastasis was detected at day 20.6 ± 1.8. The second peak occurred about 4 days after the first peak (*p* < 0.02) but there was no significant difference between the timing of the second peak and detection of metastasis. These results suggest that the second peak of activity occurs nearly simultaneous with a detectable signal from metastasis in the lungs.

Finally, an additional experiment was performed where three (n = 3) mice underwent a long-term chemical sympathectomy by administering IP injections of 6-hydroxydopamine (6-OHDA)^20,26^. 6-OHDA is known to only target catecholamine neurons via the dopamine active transport (DAT). Performing the identical inoculation, electrode implantation and recording procedures as the normal tumor-bearing mice, a significant reduction in both the overall spike activity and recorded root mean square (RMS) values were found when comparing the sympathectomized mice to normal tumor-bearing mice as shown in Table 1 which displays the overall daily activity for each experimental group.

**Table 1:** Overall daily recorded spike counts and root mean square (RMS) values per 10 min inside mammary tumor for both normal and sympathectomized mice.

Day	Tumor spike counts		Tumor activity (RMS)	
	Normal mice (n = 10)	Sympath mice (n = 3)	Normal mice (n = 10)	Sympath mice (n = 3)
13	8.27 ± 3.31	1.56 ± 1.56	0.79 ± 0.28	
14	72.10 ± 61.28	0.67 ± 0.51	0.63 ± 0.16	0.42 ± 0.08
15	42.23 ± 20.46	0.67 ± 0.33	0.60 ± 0.13	0.36 ± 0.02
16	18.10 ± 8.84	0.11 ± 0.11	0.48 ± 0.09	0.49 ± 0.10
17	8.77 ± 8.14	0.67 ± 0.51	0.60 ± 0.13	0.35 ± 0.02
18	43.45 ± 24.80	0.00 ± 0.00	0.62 ± 0.14	0.34 ± 0.01
19	79.83 ± 39.82	0.78 ± 0.22	0.62 ± 0.14	0.33 ± 0.01
20	12.88 ± 5.49	0.67 ± 0.33	0.62 ± 0.15	0.31 ± 0.02
21	5.06 ± 5.88	0.11 ± 0.11	0.51 ± 0.10	0.31 ± 0.01
22	36.65 ± 30.68	22.89 ± 22.72	0.53 ± 0.09	0.31 ± 0.02
23	3.96 ± 1.85	0.11 ± 0.11	0.49 ± 0.09	0.31 ± 0.01
24	108.64 ± 66.39	0.00 ± 0.00	0.48 ± 0.11	0.31 ± 0.00
25	19.64 ± 16.03		0.51 ± 0.12	

Sympathectomized mice show a significance decrease in overall recorded spike activity (*p* = 0.00008, two-tailed, rank-sum) and a significant decrease in overall rms activity (*p* = 0.0002, two-tailed, rank-sum) than normal tumor-bearing mice. (mean ± SEM).

### Histological confirmation of nerve fiber presence within the tumor

The experiments reported above strongly point to the presence of nerve fibers within the tumor. To confirm the presence of neural fibers within the mammary tumor mass, histology was performed on a tumor mass which was removed six days post-inoculation as shown in Fig. 5. Stains for DNA (DAPI, blue) and neurofilament (NF, in red) were performed and are shown in Fig. 5a,b. The results indicate the presence of cell bodies and the extensive network of neural fibers within the tumor mass. Figure 5a shows a large scale composite image of the two stains in a 1 mm thick tissue slice while Fig. 5b is a close-up composite image of the boxed section from Fig. 5a that clearly shows stained neural fibers that are 37.92 ± 10.75 µm (n = 12) in diameter within the tumor. At different levels of the tumor slice thickness, Fig. 5c,d also express the cancer cells (GFP) in addition to the DAPI and NF stains. Both figures show the prevalence of cancer cells within the tissue and the presence of the neural fibers embedded within.

## Discussion

In this study, we have provided evidence that electrical activity is present within mammary tumors in vivo using chronic, differential recording techniques as shown in Fig. 1. Electrode impedances were measured daily over the 12-day recording period and were found to be stable over this period and also consistent between mice (Fig. 2a). We performed acute lidocaine injection experiments directly into the tumor mass (Fig. 2b) which resulted in both a significant reduction in both the number of spike counts and the overall calculated baseline RMS of the recorded signals after injection. The lack of spike activity in the control channel prior to lidocaine injection indicates the recorded tumor activity are not noise artifacts. Furthermore, the failure to find a reduction in baseline RMS activity in the control channel, post lidocaine injection, indicates the electrical activity is contained within the tumor. Lidocaine is a well know reversible nerve block by prolonging the inactivation of the fast, voltage-gated Na^+^ channels in the neuronal cell membrane. However, using lidocaine is not solid proof the elimination of recorded activity is neural since lidocaine has also been shown to inhibit voltage-gated sodium channel, specifically Na_v_1.5, activity in colon cancer cells and reduce cancer cell invasion^27–30^.

One possible interpretation of the recorded signals is that cancer cells could generate this activity by themselves. There is a large body of evidence that electrical activity emanates from the cancer cells themselves via voltage gated ion channels. Specifically, voltage gated sodium channels (VGSC) that express channel complexes consisting of Na_v_1.5 in human MDA-MB-231 triple negative breast cancer cells. Using patch clamp techniques, inward sodium currents have been recorded which produce electrical spike activity in MDA-MB-231 cells. Using tetrodotoxin (TTX) to block these channels, a series of studies have shown a reduction in invasion^31^, potentiate a series of cell behaviors integral to the metastatic cascade^32^, VGSC activity favored the pH-dependent activity of cysteine cathepsins^33^, and found to be a central regulator of invadopodia formation which is required at the cellular level for metastasis to occur^34^. Using RNA interference method in an in vitro preparation, Na_v_1.5 was found to be primarily responsible for the VGSC-dependent enhancement of invasive behavior in MDA-MB-231 cells^35^. The effect of the beta blocker, propranolol, and VGSC blocker, ranolazine, was investigated and found that propranolol and ranolazine are functionally coupled, propranolol has direct blocking action on the VGSCs and is modulated by serum, the anti-metastatic effects of propranolol and ranolazine are not additive, and propranolol significantly decreased total Na_v_1.5 protein expression^36^.

All of the above mentioned work investigated Na_v_1.5 activity with human MDA-MB-231 triple negative breast cancer cells in vitro. The main drawback of these in vitro preparations is that they cannot replicate the tumor microenvironment and other physiological functions that could influence tumor growth and metastasis. In particular, they sever the connection to the autonomic nervous system.

The electrical activity recorded in the tumor could be generated by cancer cells expressing Na_v_1.5 as well as Na_v_1.6, K_v_10.1 and Ca_v_1.3 ion channels^37^. Our research provides strong evidence that the recorded spike activity could also be of neural origin based on three lines of evidence: (1) histology confirms the presence of nerve fibers within the tumor mass, (2) there is a direct neural connection between the peripheral tumor and the brain via the vagus nerve as shown using electrical nerve stimulation to generate evoked potentials within the tumor, and (3) chemical sympathectomy abolished the electrical activity within the tumor.

Histological analysis of the 4T1 tumor tissue and the presence of neurofilament structures within as shown in Fig. 5 clearly identifies neural fibers within the recorded tumors. In another study, researchers using the identical orthotopic 4T1 mouse model and immunohistochemical detection of tyrosine hydroxylase (TH) + found sympathetic nerves within the tumor mass and the nerves were limited to its periphery^20^.

We have shown that evoked responses within the tumor are detected when electrically stimulating the cervical vagus nerve (Fig. 2c,d). This experimental observation confirms a direct neural connection between the brain and the mammary tumor mass via the vagus nerve. The vagus contains approximately 80% afferent nerve fibers that carry sensory information from the periphery to the brain^38^ and the conduction velocity found in this experiment matches those found for mouse afferent Aδ neural fiber types primarily associated with pain^39^. Studies using mammary tumor-bearing animals with pharmacologically activated afferent vagus nerve fibers showed anti-tumoral effects in vivo and it was shown using unilateral vagotomy experiments that activation of the left vagus had a greater effect in decreasing the occurrence (or degree) of metastasis^16^. The vagus nerve effect on slowing tumor progression is thought to result from its ability to inhibit oxidative stress, inflammation and excessive sympathetic activity^40–42^. Via the production of inflammatory cytokines, the immune system can activate vagus sensory fibers that express receptors for interleukin-1 and ascend to synapses in the nucleus tractus solitarius (NTS) in the brainstem. In return, activated effector vagus nerve fibers can inhibit the production of peripheral pro-inflammatory cytokines by binding acetylcholine on tissue macrophages^43^. This negative-feedback loop system is the core of the nicotinic anti-inflammatory pathway^41^ and what is believed to give rise to the vagus tumor-protective role.

It has long been hypothesized that autonomic nerve activity, specifically the released neurotransmitters within the tumor environment, could set off a signaling pathway to promote tumor growth, or prompt new blood vessels to grow to provide the required oxygen to the tumor cells, or even alter immune cell activity to enable cancer cells to thrive unmolested. Since it has been shown that metastasis in 4T1 mice occurs around day 10 post-inoculation (Supplementary Fig. 5), the recorded electrical activity in this study results after metastasis occurs. Daily recordings and bioluminescence imaging revealed a temporally consistent electrical activity pattern within the mammary tumor mass (Fig. 3). The activity was relatively minimal except on two individual days, one roughly four days before and the other close to, or on, the day when metastasis was first detected via bioluminescence imaging (Fig. 4). This neural activity could indicate the potential interplay between neuro-endocrine activity and hypoxia, angiogenesis and cancer cell invasion and distant metastasis. Another hypothesis is the two high activity days we observed within the mammary tumor could be the sympathetic nervous system’s response to lesions forming in distant organs.

The electrical activity in the tumor was eliminated by chemical sympathectomy showing that (1) the sympathetic system is active within the tumor, and (2) the electrical activity in the tumor is coming mostly from the sympathetic fibers since it is suppressed by sympathectomy and propranolol only decreases the peak Na_v_ channel current in the tumor cells by 25% without affecting the activation and inactivation curves^36^. The significant reduction in recorded spike activity within the tumor in 6-OHDA sympathectomized mice (Table 1) is strong evidence that the activity is neural since this compound specifically ablates sympathetic nerves and markedly depletes norepinephrine (NE) in innervated organs^26,44^ and is known to upregulate sodium ion channels in other electrically excitable tissue^20,45^. Furthermore, in another study, orthotopic 4T1 mice that were treated with 6-OHDA had tumor NE concentrations that were reduced by approximately 50% suggesting that the majority of the tumor NE is derived from local sympathetic nerves. This study also showed that mice exposed to a stressor had increased NE turnover compared to non-stressed mice^20^. Under conditions that activate the sympathetic nervous system, a higher rate of NE turnover indicates greater NE utilization as defined by the process of synthesis, release, reuptake, and metabolism^46–48^. Therefore, NE turnover measured in mammary tumors is an indicator of NE synthesis and release from sympathetic nerve fibers within the tumor microenvironment^20^. These findings directly correlate with the decrease in activity which is strong evidence the activity is from sympathetic nerves within the tumor mass. Another interesting observation presented in Table 1 is the significant reduction in tumor electrical activity between normal tumor-bearing mice and sympathectomized mice. Since 6-OHDA is only known to specifically ablate sympathetic nerves, the reduced baseline RMS power could signify the reduction of VGSC activity. We, therefore, suggest that the recorded spike activity is neural and the baseline RMS power is a measure of the VGSC activity.

These results of recording chronic electrical activity directly within the peripheral tumor mass both in vivo and in situ, provides fertile ground for cancer cell biologists and neural engineers to utilize this new tool to better understand the effects of the autonomic nervous system and cellular ion channel activity on the mechanisms of specific peripheral tumor growth and metastasis. In future work, it would be advantageous to continuously record this activity starting from inoculation to capture the electrical activity in all phases of development as the mammary tumor forms, grows and metastasizes. Additionally, the potential exists to use the captured electrical signaling as a biomarker to identify ideal treatment windows for agents such as immunotherapy to patients when the cancer cells are in a compromised state.

## Methods

All surgical and experimental procedures were done with the approval and oversight of the Case Western Reserve University, Institutional Animal Care and Use Committee to ensure compliance with all federal, state and local animal welfare laws and regulations.

### Cell line and animal models

Here we developed mammary cancer metastasis using a syngeneic 4T1 orthotopic mammary fat pad mouse model. 4T1 cells were stably transfected with a lentivirus to express cytosolic firefly luciferase and green fluorescent protein (GFP) (gift from the Scheimann Laboratory at Case Western Reserve University, Cleveland, OH). 4T1 cells were cultured in RPMI medium (Gibco, Gaithersburg, MD) containing 10% fetal bovine serum (FBS) and 1% penicillin streptomycin (pen-strep). Cells were routinely tested for mycoplasma contamination to ensure cell line health and stability. 4T1 inoculations were performed using BALB/cJ mice (Jackson Laboratories, Bar Harbor, ME). Briefly, mice are anesthetized via the administration of a 2–3% isoflurane inhalant. The 9th inguinal mammary fat pad was surgically exposed and injected with an inoculant containing 0.5 × 10^6^ 4T1 cells. Meloxicam and bupivacaine were administered prior to and post-surgery, respectively. Primary tumor growth was closely monitored using bioluminescent imaging (BLI) via the luciferase tagged 4T1 cells. Previous studies have identified the dissemination metastases approximately 2 weeks after inoculation ^49–52^.

### Chronic in vivo electrode implantation procedure

Female BALB/cJ mice (6–8 weeks old, Jackson Laboratory) were used for all mammary tumor electrode implants discussed. Prior to surgery, all surgical instruments and implant assemblies were autoclave sterilized at 250º C for 1 h. During surgery, the animals were gas anesthetized under 2% isoflurane in 1L/min oxygen. Using aseptic technique, the dorsal section between the shoulder blades and the ventral abdominal area of the mouse were shaved and sterilized using alcohol and betadine scrub. A small incision was made between the shoulder blades and tunneling was done from the incision, down around the right arm and then to the ventral side abdominal area. The mouse is then placed on its dorsal side and a midline incision is made. Blunt dissection is done to expose the mammary tumor. The distal end of the DFT wire’s Teflon insulation is removed exposing 1-mm of the DTF wire. The exposed wire end is loaded into a 30-gauge hypodermic needle which is then inserted into the tumor mass. The needle is withdrawn leaving the exposed DFT wire end within the tumor. (Supplementary Fig. 1) This procedure is repeated again, inserting the second wire roughly 2-mm away from the initial wire implant. Another two DFT wires are placed under the skin on the contralateral side near the mammary fat pad. These additional two wires serve as the control signal for the experiments. A tunneling tube is then placed between the ventral incision and the dorsal incision and the electrical connector is advanced from the ventral opening to the dorsal opening. The ventral incision is then closed with 4–0 sutures (Webpro, Patterson Veterinary) and the mouse is placed on its ventral side. The electrical connector is centered between the shoulder blades and the surgical mesh base is then placed beneath the skin and sutured to the muscle fascia beneath using biodegradable 3–0 sutures (Vicryl Plus, Ethicon). A ground wire is also placed beneath the skin on the dorsal side which is comprised of a DFT wire that has 4-cm exposed wire length. The dorsal incision is then sutured closed using the same sutures as the ventral side. Mice are given two days of analgesics and rest before any tumor recordings or BLI imaging are performed.

### Implant electrode assembly process

The implant electrode assembly is comprised of five (5) electrode lead wires that are each five (5) cm long (1 × 7 × 25.4 µm: 127 µm diameter) metal-to-metal composite wire with a silver core (28%) and a stainless steel outer tubing coated with PFA insulation (35NLT-DFT wire, Fort Wayne Metals). The DFT wire insulation was stripped by approximately 2 mm at one end and soldered to an electrical connector (NCP-06-DD, Omnetics Connector Corporation). The connector was then placed in a vertical, tight-fitting, silicone tube until the pins and exposed DFT wire were inside the tube. The tube was then filled with UV curable medical grade epoxy (OG603, Epoxy Technology Inc.) until the pins and exposed DFT wires were covered and the epoxy cured. The five wires were then threaded through a small piece of surgical mesh until the mesh touched the cured epoxy. Additional UV curable epoxy was then applied to attach the surgical mesh to the assembly to form the assembly base. (Supplementary Fig. 2) The connector assembly was then removed from the silicone tube.

### Microwire electrode impedance magnitude measurements

Daily, in vivo, impedance measurements were taken between each of the four (4) microwire electrodes and the implanted ground wire using a handheld LCR meter (BK Precision, Model: 879B) and a Kelvin Clip attachment (BK Precision, Model: TL8KC1). A pre-wired Omnetics nanocircular connector (Omnetics Connector Corp, Part#: NCS-11-WD-18.0-C) was attached to the implanted percutaneous port connector and to 10kΩ resistor was placed in series to limit the current amplitude for the measurement. The meter was set to a 1 kHz sine wave and the impedance magnitude was recorded. Final impedance magnitude values were computed by subtracting the 10kΩ value from the recorded value to account for the extra series resistance.

### Chronic tumor recordings

The recording system was designed to accommodate two, differential recording channels. Each recording channel has eight amplifier hardware averaging for increased signal-to-noise recording results^53^. This was achieved by creating a custom, printed circuit board (PCB). (Supplementary Fig. 3) The PCB contains the mating connector (MCS-05-DD, Omnetics Connector Corporation) to the connector which resides in the implanted percutaneous connector. The PCB routes each differential electrode pair directly to a commercially available 16-amplifier, neural instrumentation amplifier board (C3313/RHD2216, Intan Technologies LLC). The electrode connections are routed such that one differential channel is simultaneously recorded with amplifiers 0–7 (tumor recordings) and the other is captured with amplifiers 8–15 (control recordings). The RHD2216 performs the signal filtering, analog signal multiplexing, and analog to digital conversion and is controlled via a digital SPI connection to a commercial FPGA acquisition system (Opal Kelly – XEM6010-LX45 & XEM6110 breakout board) with a custom designed PCB running Intan Technologies, LLC RHD2000 Rhythm v1.5 USB/FPGA interface software (Supplementary Fig. 4). Neural signal acquisition was performed and stored to a laptop computer using the following system configurations: 20 k samples/sec/amplifier, DSP offset removal was enabled with the high-pass filter cutoff frequency set at 100 Hz, the amplifier bandpass filters were configured to be between 100 Hz and 5 kHz.

### Post signal processing of acquired neural recordings

Using Matlab (version R2016b, Mathworks), the eight parallel amplifier channels of differential recording data from each electrode implant pair was first averaged. The data was then processed using a 7th order, zero-phase, digital bandpass filter with the lower and upper cutoff frequencies equal to 500 Hz and 1,200 Hz, respectively. The filtered data is then used to calculate the root mean squared (RMS) value of the recorded electrogram waveforms. The threshold value is determined by taking the RMS value and multiplying it by six to convert from RMS to a peak-to-peak voltage value. Since we are interested in positive value thresholds this value is divided in half. A factor of 3.5 was applied to the threshold value to add margin from falsely detecting spurious noise that is just above the baseline noise value. The resulting threshold levels were typically around 10 µV based on the electrode impedance and recording system noise. The ENG waveforms were then processed using a MATLAB algorithm that uses fuzzy c-means clustering (*fcm*) to threshold and count spikes^54,55^. This algorithm first identifies spikes as local maxima that exceed a user-specified threshold. These spikes are then aligned with the maxima centered and 1.5 ms (30 samples) of data before and after the maximum saved for analysis. To avoid the false positive spike counts, the spikes from the control site and tumor site which occur at the same time were eliminated from the resulting spike count number.

### Lidocaine injection to eliminate neural activity

To test the effect of nerve blocking agent on neural tissue in the tumor, terminal lidocaine experiments were performed (n = 5). Lidocaine blocks the voltage gated sodium channels thereby preventing the nerve from initiating an action potential. While the mouse was under anesthesia (2% Isoflurane in 1L/min oxygen), 0.5 ml of 2% lidocaine hydrochloride solution is injected directly into the tumor, on the first peak day of neural activity. Neural activity is recorded for 10 min immediately before and after the injection. Mice were immediately perfused after termination of the recording procedure.

### Simultaneous vagus nerve stimulation and tumor recording

Under anesthesia (2% Isoflurane in 1L/min oxygen), two BALB/cJ mice that were both 17-days post-inoculation underwent the following procedure. While lying on their dorsal side, the right cervical vagus nerve was exposed and freed from connective tissue and the carotid artery. Two hook electrodes made from PFA-coated Tungsten rods 200 µm in diameter (A-M Systems, part#:718,500). The PFA was removed at both ends and the distal end was bent to form a hook which the vagus nerve was then placed on. The proximal end had insulated wires soldered to it and connected to a current controlled stimulation unit (AlphaOmega – AlphaLab SnR). An incision in the skin was made on the ventral side near the tumor. Blunt dissection was done to expose the tumor and two microwire electrodes were implanted within the tumor mass and connected to the recording electronics as described above. While recoding from the tumor electrodes, repeated monophasic current pulses were delivered to the vagus nerve (0.25 Hz, 100 µs pulse width, 6 mA amplitude). Post-processing of the recorded data was performed in Matlab (version R2016b) to calculate the propagation time from the stimulus onset to the peak amplitude response of the evoked potential in the tumor mass. The mice were perfused right after the experiment concluded.

### Chemical sympathectomy

6-hydroxydopamine (6-OHDA, 100 mg/kg body weight) dissolved in sterile saline containing 0.01% ascorbate was immediately administered intraperitoneally (IP), 4 and 2 days prior to electrode implantation (n = 3) and thereafter every five (5) days to prevent reinnervation and maintain a long-term sympathectomy^20,26^. The mice then underwent the implant surgery and chronic recording procedures described above.

### Bioluminescent imaging

Bioluminescent Imaging (BLI) was performed using an IVIS Spectrum Imaging System (Perkin Elmer, Waltham, MA). Briefly, BLI was performed ~ 10 min after an intraperitoneal injection of 200 μL of D-Luciferin (12.5 mg/mL). Imaging was performed daily to closely monitor metastatic development in the thoracic cavity of tumor bearing mice.

### Histology

The mouse was placed under anesthesia (3% isoflurane) and two lateral and two anterio-medial incisions on the ventral side, the thoracic cavity viscera was exposed. The body tissue was fixed with 20 mL, 1X, Phosphate Buffered Saline (PBS) Solution followed by 20 mL, 4%, Paraformaldehyde (PFA) solution injected via the heart. The transected tumor tissue was transferred to PBS for 24–48 h. The tissue was then cut into 1 mm thick slices and then washed in solution containing 80% Methanol and 20% Ethanol overnight. Slices were then treated with a blocking buffer solution for one hour at 37 °C. The blocking buffer consisted of 1–3% BSA, 0.01% NaN3 & 0.5% Triton-X in PBS. The tissue was then stained with 1° antibody i.e. Neurofilament antibody (N4142, Sigma Aldrich, USA) by washing it with a mixture of antibody and blocking buffer solution at 37 °C overnight. The antibody-to-blocking buffer solution concentration used was a 1:200 ratio, respectively. Similar procedures were followed for staining with 2° antibody i.e. Alexa-Fluor 594 (A32754, ThermoFisher Scientific, USA) simultaneously with DAPI. The cells were marked with Green Fluorescent Protein (GFP) marker while they were being cultured. 4′,6-diamidino-2-phenylindole (DAPI) stain was used to mark the nuclei of the tumor cells. Images were captured for both GFP and DAPI stains as well to understand the extent of the innervation into tumor cells. Slices were then washed three times with Phosphate Buffered Saline with Triton (PBST) with an interval of ten minutes each. PBST consisted of 0.5% TritonX in PBS. Finally, the slices were introduced into tissue clearing solution, based on the technique lipid-preserving index matching for prolonged imaging depth (LIMPID)^56^. Cleared tissue was then transferred to imaging disc in the medium of mineral oil. Slices were imaged using Leica HyVolution SP8 confocal microscope.

## Supplementary information

Supplementary Information.

Supplementary Video.
