# Supplementary material for: Chronic neural activity recorded within breast tumors
Source: Sci Rep. 2020 Sep 9;10:14824. doi: 10.1038/s41598-020-71670-y (PMC7481786; doi:10.1038/s41598-020-71670-y)
Supplement: Supplementary file 1 — Supplementary Information. [file 41598_2020_71670_MOESM1_ESM.pdf]

Chronic neural activity recorded within breast tumors

Grant A. McCallum[1]*, Jay Shiralkar[1], Diana Suciu[1], Gil Covarrubias[1,2], Jennifer S. Yu[2,3,4], Efstathios Karathanasis[1,2], Dominique M. Durand[1]*

[1]Department of Biomedical Engineering, Case Western Reserve University, Cleveland, Ohio, USA. [2]Case Comprehensive Cancer Center, Case Western Reserve University, Cleveland, Ohio, USA. [3]Department of Radiation Oncology, Taussig Cancer Institute, Cleveland Clinic, Cleveland, Ohio, USA. [4]Department of Cancer Biology, Lerner Research Institute, Cleveland Clinic, Cleveland, Ohio, USA. *Corresponding author. Email: gam19@case.edu (G.M.); dxd6@case.edu (D.D.)

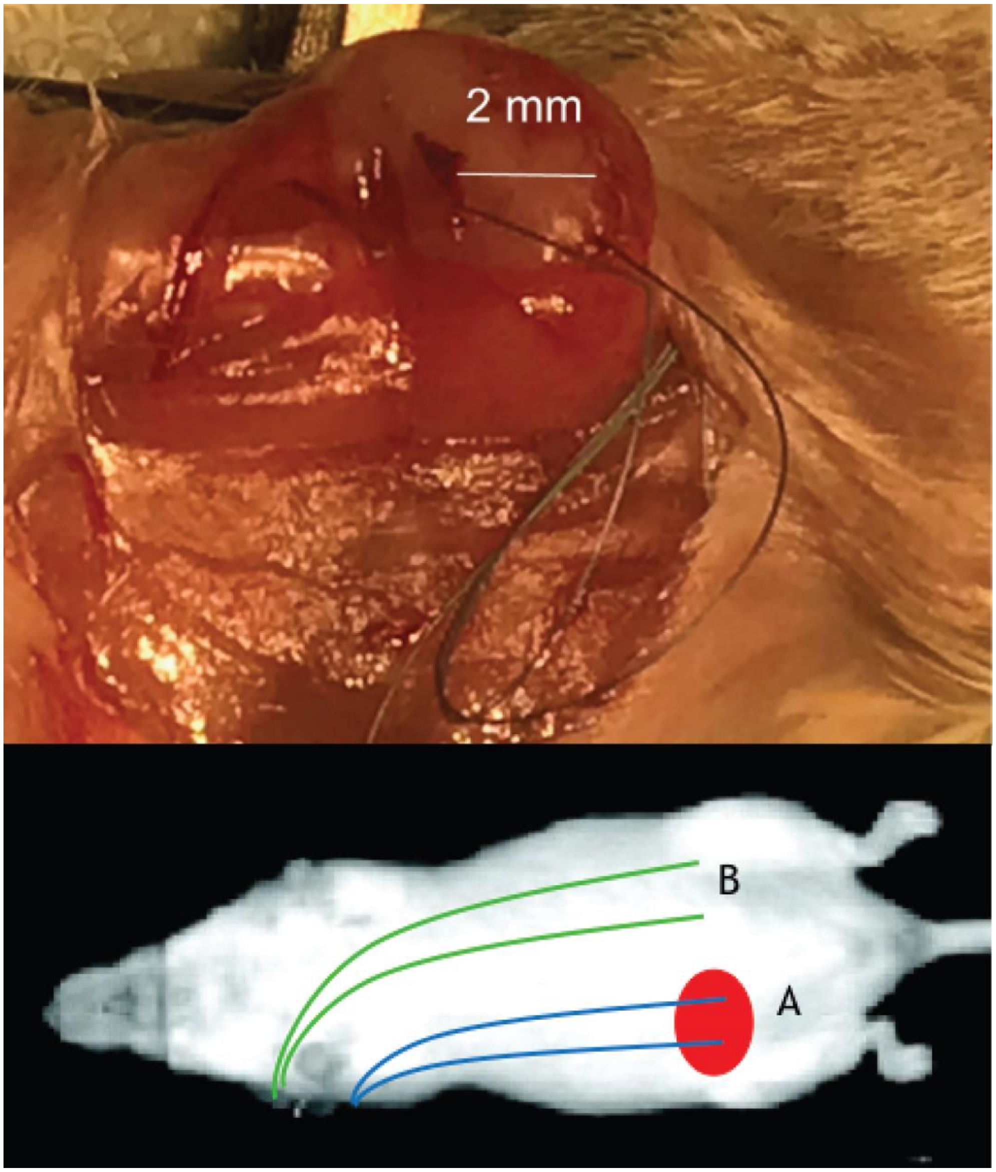

Supplementary Figure 1

Microwire electrode implantation and subcutaneous lead placement. (**Top**) Two microwire electrodes are surgically implanted into the breast tumor mass at approximately day 10 post-inoculation. The two electrodes are implanted directly in the tumor approximately 2 mm apart. (**Bottom**) A total of four microwire electrodes, two for the tumor (A) and another two on the contralateral side and act as a control signal (B), are implanted in each animal. All four microwire electrodes are insulated with PFA except for a 1 mm exposed distal end. The proximal ends of all four electrode leads are soldered to a percutaneous connector prior to surgery and tunneled subcutaneously to the animal's dorsal side.

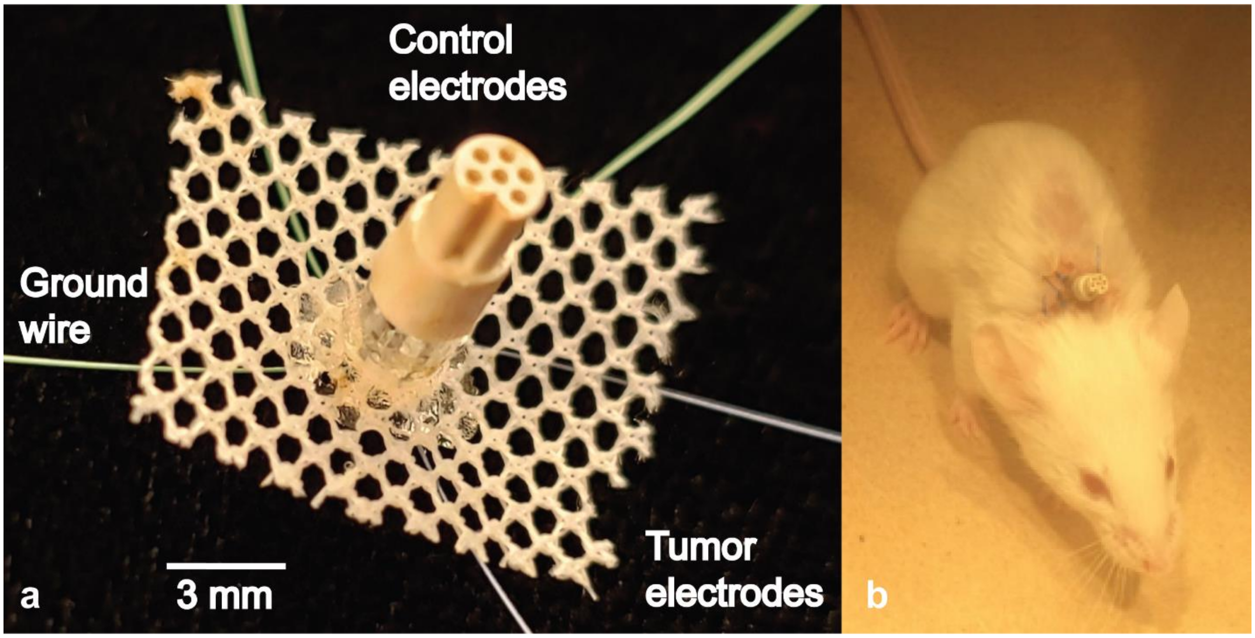

Supplementary Figure 2

Percutaneous connector assembly and implant for chronic neural recordings within tumor. (**a**) Fabricated percutaneous connector assembly prior to implant showing the two tumor electrode leads (blue), the two control electrode leads (green) and the ground wire lead are all soldered to a circular electrical connector (A79108-001, Omnetics Connector Corporation, USA) and insulated with biocompatible, UV curable epoxy and fixed to a surgical mesh. (**b**) Post-operative percutaneous connector implant showing the connector assembly successfully placed on the animal's dorsal side.

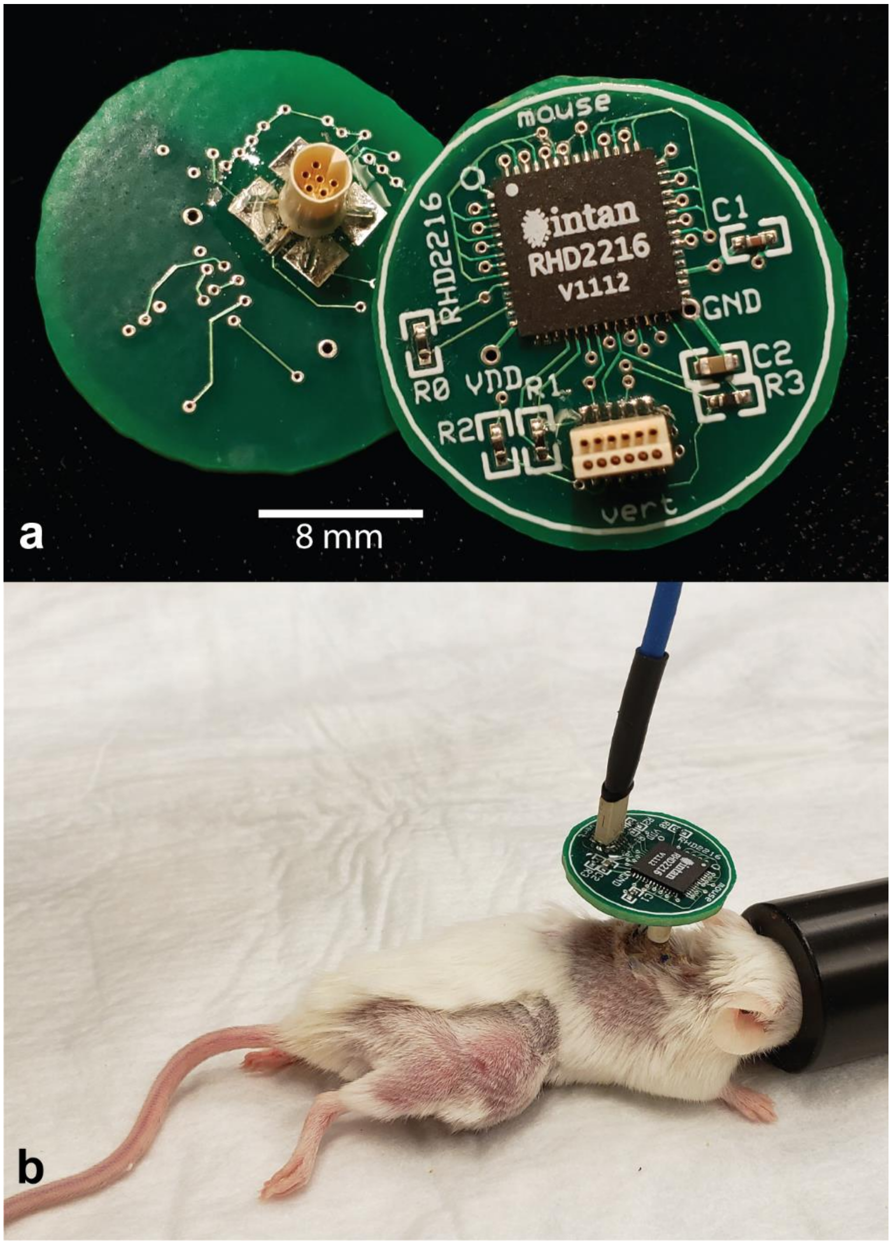

Supplementary Figure 3

Custom neural amplifier board for chronic tumor recordings and anesthetized recording session of tumor bearing mouse. (**a**) Displaying the PCB backside with the 6-pin Omnetics circular connector that directly mates to the percutaneous port implanted in the mouse. The PCB front-side contains the Intan Technologies, LLC RHD2116 neural amplifier device, passive components and a 12-pin, nano polarized connector from Omnetics for a SPI cable attachment. (**b**) Neural amplifier board is connected to an anesthetized mouse and a SPI cable is connected from the amplifier board to a FPGA data acquisition board to collect and store the recorded neural data to a laptop computer.

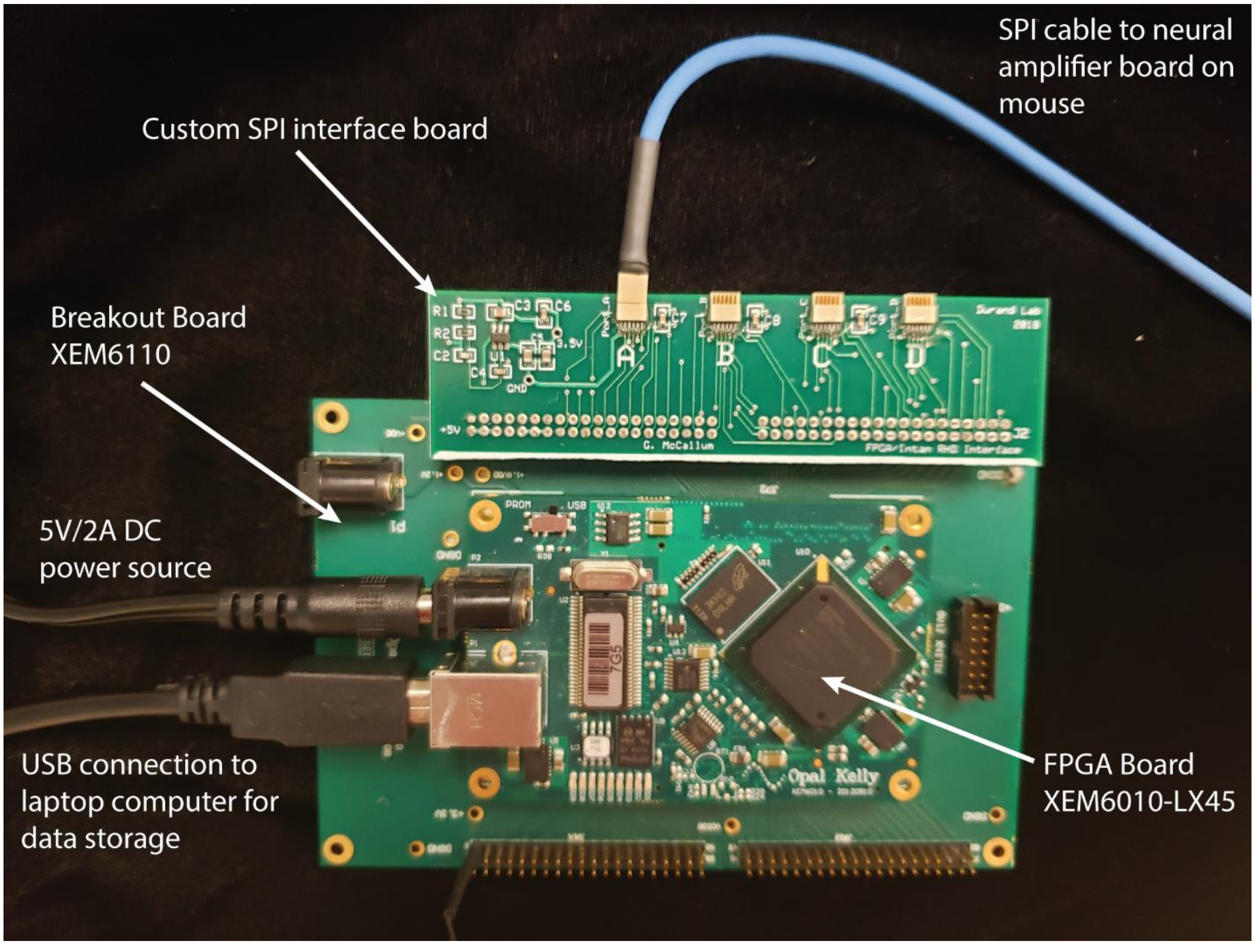

Supplementary Figure 4
FPGA data acquisition board to collect and store recorded neural data from the implanted tumor and control electrodes. Digitized and multiplexed neural recording data from both the tumor and the control electrodes are sent over the SPI interface cable from the neural amplifier board connected to the mouse. The SPI cable is connected to a custom designed and built interface board which is mounted on a commercial, off the shelf breakout and FPGA board from Opal Kelly Incorporated. The FGPA is programmed with firmware created and freely downloaded from Intan Technologies, LLC. The FPGA board collects the recorded neural data and manages its transfer to a laptop computer via a USB connection.

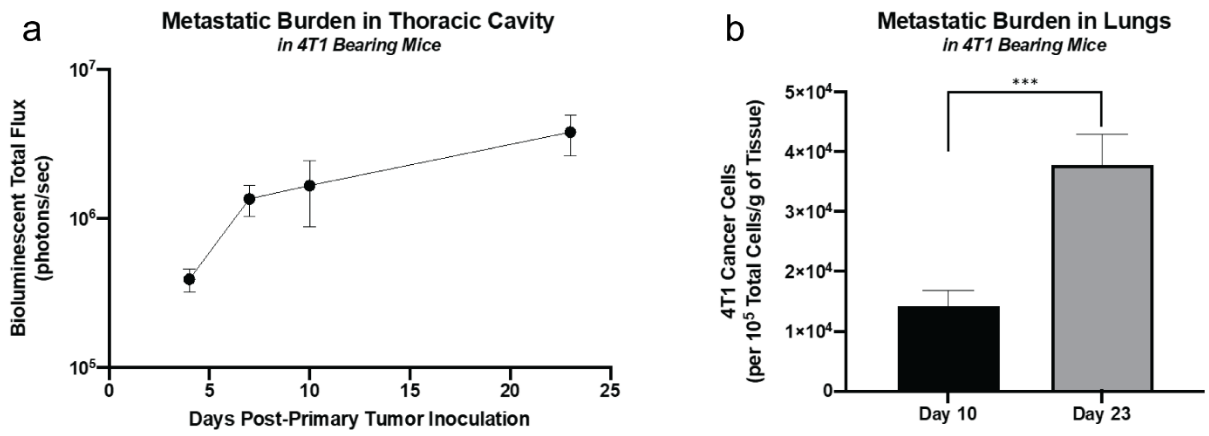

Supplementary Figure 5

Measuring metastatic burden in the thoracic cavity and lungs over time using bioluminescence imaging and flow cytometry analysis. (**a**) The bioluminscent signal of the thoracic cavity is recorded and displayed as a progression over time to monitor 4T1 cell expansion. Data is represented as mean ± SEM (n=4). (**b**) Using the 4T1 tumor model, mice were allowed to develop spontaneous metastasis. The metastatic outgrowth within lung tissue was analyzed at multiple time points; day 10 and 23. At day 10, mice were euthanized and their lungs were excised. The lung tissue was gently disrupted and developed into a single cell suspension for flow cytometry analysis. Using the GFP probe associated with the 4T1 cancer cells, a quantitative value for the total number of cancer cells within lung tissue was recorded. The total number of 4T1 cancer cells were normalized via the total viable cells and per gram of lung tissue. Similar procedures were implemented for day 23 data. Data is represented as mean ± SEM (n=10, day 10 and n=10, day 23).

Supplementary Movie 1

Video and audio representation of the neural spike activity with a mouse breast tumor. Neural activity recorded within a mouse breast tumor was converted to an audio signal and synchronized with the electroneurogram (ENG) waveform to produce a 30 second movie of the neural spiking activity that occurs within the tumor mass.
